# Perceptions and Attitudes Toward Research Participation Among Medical Students at an Irish Medical School - A Cross-Sectional Study

**DOI:** 10.1177/23821205261466057

**Published:** 2026-08-28

**Authors:** Shobha Mehta, Padraig Cronin, Hanru Dong, Maeve Boyle, Mark G. Rae, Mohammed H. Abdulla

**Affiliations:** 1 School of Medicine, 8795 University College Cork, Cork, Ireland; 2 Medical Research & Technology Society, 8795 University College Cork, Cork, Ireland; 3 Department of Physiology, 8795 University College Cork, Cork, Ireland

**Keywords:** medical student research, research barriers, student perceptions, mentorship, medical education

## Abstract

**Introduction:**

Participation in research enables medical students to develop critical appraisal, communication, problem-solving, and analytical skills. As research engagement is increasingly important for medical education and progression into postgraduate training pathways, this study aimed to assess medical students’ perceptions of research, identify perceived barriers to participation, and compare attitudes across student cohorts.

**Methods:**

Medical students at University College Cork were invited to complete an online questionnaire via the Qualtrics platform. The survey included multiple-choice, open-text, and Likert-scale questions assessing the perceived importance of research and barriers to participation. Data were processed and analysed using Microsoft Excel and SPSS, respectively. Frequency analyses and chi-squared tests were conducted to examine differences between student cohorts.

**Results:**

A total of 114 students participated, including 60.5% from the EU and 39.5% from outside the EU. Overall, 82.4% of respondents considered research important to their medical education, including 82.6% of EU students and 82.2% of non-EU students. Additionally, 81.4% considered research important for their future careers. The most commonly reported barriers to research participation were limited time (77.2%) and limited research knowledge (75.3%). No significant differences were observed between EU and non-EU students in perceptions of research importance.

**Conclusions:**

This study highlights the perceived importance of research by medical students, whilst identifying significant barriers such as limited research knowledge and insufficient mentorship hindering their participation. Concerted efforts to address these challenges may foster greater student engagement in research, ultimately benefiting their academic development and career progression.

## Introduction

Participation in research is becoming an increasingly important aspect of medical students’ education, and research activities are frequently highlighted as assets in applications to medical school, internships and residencies. Indeed, research experience increases the likelihood of residency match success.^1-3^ Participating in scholarly research allows students to develop their abilities in critically evaluating research findings, communicating findings to their peers and patients, and developing problem-solving and analytical skills.

The importance of research experience is reflected in the fact that a number of medical schools have made research skills development a mandatory part of their curricula.^4-7^ Notably, the Royal College of Physicians and Surgeons of Canada have included the role of the scholar in their Canadian Medical Education Directives for Specialists (CanMEDS) framework, defining scholar as a physician who critically evaluates information and its sources, and contributes to the creation, dissemination, application and translation of medical knowledge.^8^ Furthermore, involvement in research as a medical student increases the likelihood of those individuals going on to pursue an academically focused career, with ongoing research and publication activities.^6,9,10^ Despite the clear personal and professional benefits to participating in research, students without formal research experience often experience anxiety, hesitancy and uncertainty when attempting to enter the research sphere.^11,12^ These observations underscore the need for early, structured research training within medical education to build confidence and foster sustained academic engagement.

Previous studies have attempted to characterise medical students’ attitudes, perceptions and perceived barriers to engaging in research. A cross-sectional study of Australian medical students found that 449 of 704 (63.8%) of respondents identified uncertainty surrounding how to find research opportunities as a barrier to their participation.^13^ A literature review of medical student experiences, attitudes and outcomes with scholarly research identified time constraints, lack of acknowledgement and lack of mentorship as barriers to research experience.^4^ Similarly, a systematic review and meta-analysis of medical students’ perspectives on engagement in research identified lack of time as a primary barrier.^14^ In addition, lack of resources or student interest was identified as a barrier in a cross-sectional study of 215 students in Abbottabad, Pakistan.^15^ A cross-sectional study of medical students in Riyadh found that a significant barrier to research participation was inadequate supervisor guidance and support.^16^ Another cross-sectional study of 133 students in Colombia identified time restrictions (71%), lack of meaningful mentorship (30%) and financial constraints (20%) as barriers to research participation.^17^ One of the largest studies collected responses from 1625 medical students from 38 countries and again identified lack of time and difficulty finding mentors or projects as key perceived barriers.^18^ Together, these studies identified key barriers to medical students’ participation in research across diverse international settings, which include a lack of time, insufficient mentorship or supervisor support, difficulty finding research opportunities, limited resources, and financial constraints.

University College Cork (UCC) offers both a five-year direct entry program for students entering directly from secondary school and a four-year graduate entry program for students with at least a bachelor ‘s-level prior education. Until recently, a mandatory course component required students to complete a final year research project, which involved securing a research supervisor, producing a topic of interest and study proposal, securing ethical approval where required, and analysing and presenting their project results. However, this component was made optional in 2024 for all incoming classes, leaving no formal requirement for primary research as part of the degree. This study aimed primarily to evaluate medical students’ perceptions of research engagement at an Irish university, specifically within a cohort of third-level students. Secondly, the study aimed to explore the barriers students face in engaging with medical research and to compare attitudes across different student groups, with a particular focus on possible distinctions between European Union (EU) and non-EU students.

## Materials and Methods

This study took the form of a Cross-Sectional Analysis performed at a single centre. Ethical approval was obtained from the University College Cork Social Research Ethics Committee during the summer of 2024, before the collection and analysis of data. The population of interest was medical students enrolled at UCC in either the direct or graduate entry program, from any year of study. No limitations were placed on sex, primary language, or prior education. An age restriction of ≥ 18 years was applied.

Recruitment took place over two phases, with initial capture being performed between August and December 2024, followed by a second capture to improve population size between November 2025 and January 2026. Participants were recruited through a variety of methods, including pre-existing email lists as part of the MRT Society, as well as requests for the circulation of the survey *via* email by the UCC Medical School Research department. Links to the study were also circulated *via* social media, a primary form of communication for UCC societies. No incentive items were offered during the recruitment process as per ethical approval.

An electronic-based questionnaire was designed using a combination of multiple choice, open text responses, and five-point Likert-type statements ranging from “strongly disagree” to “strongly agree”, modeled after the validated Medical Student Scholar-Ideal Mentor Scale (MSS-IMS).^19^ The use of the MSS-IMS was included as part of a larger study to examine longitudinal aspects of research mentorship and thus is outside the scope of this paper. Information was also collected on demographics and previous research experience with question development being performed in close collaboration with two independent academic supervisors (MA, MR). Questionnaire responses, both selected responses and free text, were coded to a numeric scale for statistical analysis (Appendix 1). The questionnaire was constructed and implemented using the Qualtrics platform.

Examination of the data involved a two-step process beginning with the transfer of the data from the Qualtrics Cloud Server to a local Excel Spreadsheet (Software Version 2410), where the responses were organised and processed to remove responses which did not meet the inclusion criteria. Likert scale responses which were left incomplete were removed from the dataset for individual analysis to avoid biasing the results, with final sample sizes reported in the results (Tables 2 and 4). Once organised, the data were transferred to the SPSS Statistical Software Package (Software Version 29.0.2.1) and analysed using the in-built Frequency Analysis to examine individual variable characteristics, as well as Chi-Square testing to examine relationships between the variables. In cases where the Expected Cell Count Assumption was not met for a Chi-Square test value, the Likelihood Ratio was used to assess relationships between variables. Significance was determined using a p-value < 0.05. Overall preparation of the study manuscript was completed in accordance with the STROBE Reporting Checklist of Cross-Sectional Studies (Appendix 2) in line with quantitative studies in epidemiological research.^20^

## Results

**Figure 1.**
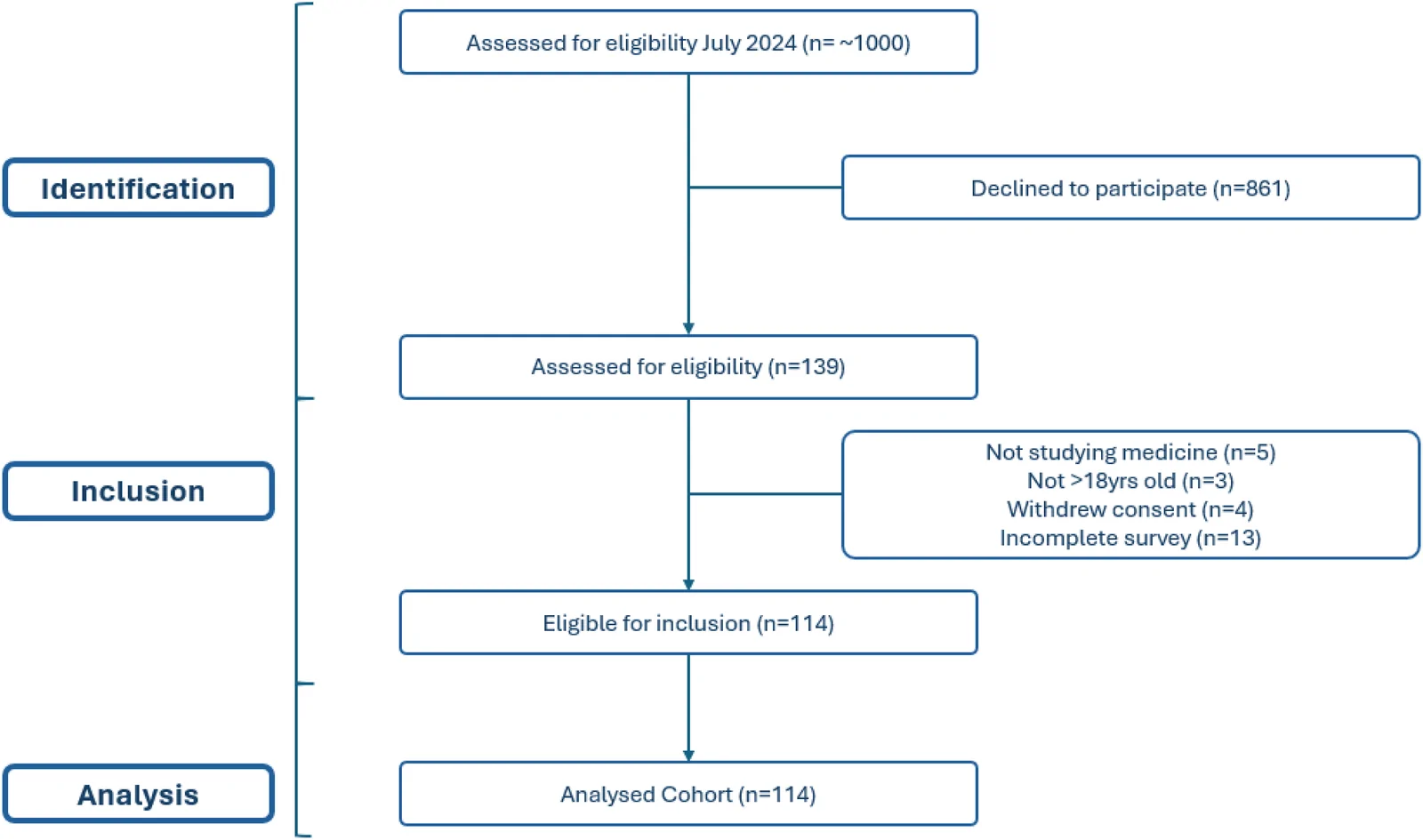
Study design and recruitment pathway as illustrated by the STROBE flowchart

Following completion of data analysis, a cohort of 114 students was examined in this study with their associated academic backgrounds (Figure 1). Assuming a medical student population of approximately 1,000 students, a sample size of 278 respondents would be required to estimate proportions with a 95% confidence level and a 5% margin of error. A total of 114 responses were obtained, corresponding to an estimated margin of error of approximately ±8.4%.

This study’s demographics (Table 1) showed a higher proportion of female than male respondents (66.7%) and a higher proportion of direct entry program students (68.4%), which reflects the current programme sizes (approximately 120 direct entry students and 80 graduate entry students per academic year). A higher proportion of EU students (60.5%) than non-EU students (39.5%) participated in the study and thus had greater representation in this study population.

**Table 1.** Descriptive Breakdown of the Study Cohort, Including Gender, Programme of Study, and Nationality of Participants

Variable	Sub variable	Proportion of cohort
Gender (*n=114*)	Male	36 (31.6%)
	Female	76 (66.7%)
	Prefer not to say	2 (1.8%)
Entry Route	Direct Entry	78 (68.4%)
	Graduate Entry	36 (31.6%)
Nationality	EU	69 (60.5%)
	Non-EU	45 (39.5%)
Year of Study	Direct Entry - Year 1	25 (21.9%)
	Direct Entry - Year 2	16 (14.0%)
	Direct Entry - Year 3	23 (20.2%)
	Direct Entry - Year 4	11 (9.6%)
	Direct Entry - Year 5	3 (2.6%)
	Graduate Entry - Year 1	4 (3.5%)
	Graduate Entry - Year 2	21 (18.4%)
	Graduate Entry - Year 3	9 (7.9%)
	Graduate Entry - Year 4	2 (1.8%)
Prior Research Experience	Yes	53 (46.5%)
	No	61 (53.5%)
Prior Research Publication	Yes	29 (25.4%)
	No	85 (74.6%)

Responses to questions regarding research participation are summarised for all students in Table 2, and a comparison between EU and non-EU students is summarised in Table 3. Overall, respondents agreed that research is an important component of the medical curriculum, with similar proportions of EU students (82.6%) and non-EU students (82.2%) agreeing (Table 3). Additionally, 81.4% of students viewed research as important for their future careers (47.8% strongly, 33.6% somewhat). When exploring the obligation to participate in medical research, there was a significant difference between EU and non-EU students. Specifically, 57.8% of non-EU students strongly agreed that they felt obliged to participate in research, while only 24.6% of EU students felt the same (*LR = 17.781, p = 0.001*). Non-EU students were more likely to view research participation as essential for career progression, with 86.7% agreeing (68.9% strongly, 17.8% somewhat), compared to just 58.9% of EU students, with 23.5% of EU students expressing neither agree nor disagree and 17.6% disagreeing to some extent (*LR = 22*.*634, p<0.001*). Intentions to continue research after medical school were broadly similar across groups. For instance, among EU students, 65.2% agreed (28.8% strongly, 36.4% somewhat), and among non-EU students, 66.7% agreed (42.9% strongly, 23.8% somewhat).

**Table 2.** Response Data for Questions Examining the Perceptions of Students to Medical Research

​	Strongly disagree	Somewhat disagree	Neither agree nor disagree	Somewhat agree	Strongly agree	No. of responses
“Research is an important part of the medical curriculum”	1 (0.9%)	5 (4.4%)	14 (12.3%)	43 (37.7%)	51 (44.7%)	114
“Research is an important part of my future career”	1 (0.9%)	6 (5.3%)	14 (12.4%)	38 (33.6%)	54 (47.8%)	113
“I feel obligated to participate in research activities”	1 (0.9%)	11 (10.0%)	18 (16.4%)	38 (34.5%)	42 (38.2%)	110
“Research Experience is necessary for my application to internship or residency”	3 (2.7%)	12 (10.6%)	19 (16.8%)	30 (26.5%)	49 (43.4%)	113
“I have a genuine interest in research activities”	7 (6.6%)	22 (20.8%)	18 (17.0%)	31 (29.2%)	28 (26.4%)	106
“I expect to continue research activities beyond medical school”	4 (3.7%)	10 (9.3%)	23 (21.3%)	34 (31.5%)	37 (34.3%)	108

**Table 3.** Response Data Comparing the Perceptions of EU vs Non-EU Students to Medical Research

Question	Response proportion					EU vs Non-EU	
	Strongly disagree	Somewhat disagree	Neither agree nor disagree	Somewhat agree	Strongly agree	Likelihood ratio	Significance
“Research is an important part of the medical curriculum” (EU)	0 (0%)	4 (5.8%)	8 (11.6%)	25 (36.2%)	32 (46.4%)	3.0050	0.5570
“Research is an important part of the medical curriculum” (Non-EU)	1 (2.2%)	1 (2.2%)	6 (13.3%)	18 (40%)	19 (42.2%)		
“Research is an important part of my future career” (EU)	0 (0%)	4 (5.9%)	9 (13.2%)	28 (41.2%)	27 (39.7%)	7.388	0.117
“Research is an important part of my future career” (Non-EU)	1 (2.2%)	2 (4.4%)	5 (11.1%)	10 (22.22%)	27 (60%)		
“I feel obligated to participate in research activities” (EU)	1 (1.5%)	10 (15.4%)	10 (15.4%)	28 (43.1%)	16 (24.6%)	**17.781**	**0.001**
“I feel obligated to participate in research activities” (Non-EU)	0 (0%)	1 (2.2%)	8 (17.8%)	10 (22.2%)	26 (57.8%)		
“Research Experience is necessary for my application to internship or residency” (EU)	3 (4.4%)	9 (13.2%)	16 (23.5%)	22 (32.4%)	18 (26.5%)	**22.634**	**<0.001**
“Research Experience is necessary for my application to internship or residency” (Non-EU)	0 (0%)	3 (6.7%)	3 (6.7%)	8 (17.8%)	31 (68.9%)		
“I have a genuine interest in research activities” (EU)	3 (4.7%)	16 (25.0%)	11 (17.2%)	18 (28.1%)	16 (25.0%)	2.554	0.768
“I have a genuine interest in research activities” (Non-EU)	4 (9.5%)	6 (14.3%)	7 (16.7%)	13 (31%)	12 (28.6%)		
“I expect to continue research activities beyond medical school” (EU)	2 (3%)	7 (10.6%)	14 (21.2%)	24 (36.4%)	19 (28.8%)	3.618	0.606
“I expect to continue research activities beyond medical school” (Non-EU)	2 (4.8%)	3 (7.1%)	9 (21.4%)	10 (23.8%)	18 (42.9%)		

The most highly reported barrier to research by the respondents was limited time (Table 4). For EU students, this was the most strongly affirmed barrier, with 78.8% agreeing (40.9% strongly, 37.9% somewhat) and only 6.1% disagreeing. Non-EU students also recognized this issue, with 75% agreeing overall (50% strongly, 25% somewhat), though they showed slightly more distribution across the response spectrum. Limited research opportunities were more strongly perceived as a barrier by non-EU students. The majority (67.5%) of non-EU respondents agreed that limited opportunities impeded their ability to engage in research, compared to only 51.6% of EU students. EU students presented a more even spread of responses, with 31.8% neither agreeing nor disagreeing and a total of 51.6% in agreement (25.8% strongly, 25.8% somewhat).

**Table 4.** Response Data for Questions Examining the Perceptions of Barriers to Research Experience

Question	Response proportion					
	Strongly disagree	Somewhat disagree	Neither agree nor disagree	Somewhat agree	Strongly agree	No. of responses
Limited Finance	5 (5.1%)	13 (13.3%)	32 (32.7%)	34 (34.7%)	14 (14.3%)	98
Limited Supervision/Mentorship	2 (1.8%)	10 (9.1%)	20 (18.2%)	43 (39.1%)	35 (31.8%)	110
Limited Research Opportunities	1 (0.9%)	16 (14.7%)	29 (26.8%)	35 (32.1%)	28 (25.7%)	109
Limited Research Knowledge	4 (3.7%)	9 (8.3%)	14 (12.8%)	33 (30.3%)	49 (45%)	109
Limited Time	0 (0.0%)	6 (5.5%)	19 (17.3%)	36 (32.7%)	49 (44.5%)	110

Additionally, a lack of research knowledge was reported by the majority of students as an impediment to research activity, with 74.7% of EU students and 76.2% of non-EU students agreeing. Limited supervision or mentorship was also identified as a prominent barrier, with a similar degree of agreement between cohorts (71.2% among EU students and 70.4% among non-EU students).

Finally, financial limitations were also perceived differently between EU and non-EU students, with 55.1% (17.2% strongly, 37.9% somewhat) of EU students *versus* only 40% (10% strongly, 30% somewhat) of non-EU students, agreeing that a lack of finances impacted their ability to conduct research (Table 5).

**Table 5.** Response Data for Questions Comparing the Barriers of EU vs Non-EU Students to Medical Research

Question	Response proportion					EU vs Non-EU	
	Strongly disagree	Somewhat disagree	Neither agree nor disagree	Somewhat agree	Strongly agree	Likelihood ratio	Significance
Limited Finances (EU)	2 (3.4%)	7 (12.1%)	17 (29.3%)	22 (37.9%)	10 (17.2%)	3.26	0.66
Limited Finances (Non-EU)	3 (7.5%)	6 (15%)	15 (37.5%)	12 (30%)	4 (10%)		
Limited Supervision/Mentorship (EU)	1 (1.5%)	7 (10.6%)	11 (16.7%)	29 (43.9%)	18 (27.3%)	2.789	0.594
Limited Supervision/Mentorship (Non-EU)	1 (2.3%)	3 (6.8%)	9 (20.5%)	14 (31.8%)	17 (38.6%)		
Limited Research Opportunities (EU)	1 (1.5%)	10 (15.2%)	21 (31.8%)	17 (25.8%)	17 (25.8%)	4.872	0.301
Limited Research Opportunities (Non-EU)	0 (0%)	6 (14%)	8 (18.6%)	18 (41.9%)	11 (25.6%)		
Limited Research Knowledge (EU)	3 (4.5%)	5 (7.5%)	9 (13.4%)	18 (26.9%)	32 (47.8%)	1.471	0.832
Limited Research Knowledge (Non-EU)	1 (2.4%)	4 (9.5%)	5 (11.9%)	15 (35.7%)	17 (40.5%)		
Limited Time (EU)	0 (0%)	4 (6.1%)	10 (15.2%)	25 (37.9%)	27 (40.9%)	2.404	0.493
Limited Time (Non-EU)	0 (0%)	2 (4.5%)	9 (20.5%)	11 (25%)	22 (50%)		

## Discussion

To the best of our knowledge, this is the first study that has analysed the perceptions of medical students at an Irish medical school towards medical research. The higher proportion of female respondents in this study is reflective of current trends in medical education and the increasing number of women entering the medical profession.^21,22^ The higher proportion of direct-entry students who participated in the study is also in line with current statistics in Irish medical education with graduate entry students still being in the minority.^23^

The vast majority of students agreed with the statements that research was an important part of the medical curriculum and their future careers, in keeping with current curricula priorities. In addition, over 70% of respondents endorsed feeling obliged to participate in research activities. Of those respondents, a statistically significantly higher proportion of non-EU students strongly agreed that they felt obliged to participate in research activities (57.8%), compared to only 24.6% of EU students. While this study did not capture the reasoning behind these differences, there are a number of factors that may help to explain them. It is possible that non-EU students experience a greater degree of external pressure to conduct research, possibly due to their perception that it is a requirement of their medical training, compared to EU students, the majority of whom are Irish. Irish students, who may be more familiar with, or reliant upon, local or European training systems that place comparatively less emphasis on undergraduate research credentials, may approach research engagement with more variability in motivation. Although overall agreement was similar between cohorts, non-EU students were more likely to strongly agree that research is important for their future careers than their EU counterparts (42.9% vs 28.8%). For many non-EU students, especially those seeking postgraduate training abroad or aiming to return to highly competitive medical systems in their home countries, participation in research is often viewed as a critical differentiator in residency and internship applications.^24^ The responses of the EU cohort suggest a broader spectrum of personal interest rather than academic pressure in training, although further research is needed to confirm this hypothesis. This distinction highlights the need for support systems that acknowledge differing motivations, whilst offering career-aligned pathways for those seeking research opportunities.

While respondents clearly demonstrated an understanding of the importance of research participation, they also identified several barriers that hindered their participation. Despite not meeting the standard for statistical significance in this study, the pattern of these responses provides insights into potential challenges faced by medical students engaging in research. The most commonly identified barriers among all respondents were limited mentorship (70.9%), limited research knowledge (75.3%) and limited time (77.2%), findings which are in line with previous studies among similar cohorts.^4,13-18^ There were no statistically significant differences in barriers to research participation that were identified between EU and non-EU groups. These findings suggest the need for improved mentorship resources in medical education and could inform the development of future initiatives supporting this. While guidance from experienced researchers is invaluable for the development of students’ research abilities, a more accessible form of mentorship from their peers may empower students to approach their first research activity, gain confidence and ease their progression into a more structured research experience. Being able to ask questions in a supportive environment also allows for improved research knowledge, with more room for trial and error than a formal research relationship.

These data also suggest that there may be gaps in formal training around research methodology, critical appraisal, and academic writing within the medical curriculum. Such gaps could contribute to a lack of confidence or readiness to pursue research opportunities independently.^25^ This underscores the need to embed structured research education into undergraduate medical programs in order to ensure that students are given the tools and training to apply their knowledge meaningfully. The integration of such peer mentorship with structured research activities provided by medical schools and institutions would allow for increased participation, positive experiences and decreased anxiety among medical students who clearly recognize the importance of engaging in research for their future careers.

Most students also identified limited time as a key barrier to conducting research. Notably, stronger agreement was more common among non-EU compared to EU (50% vs 40.9%). This disparity might reflect unique demands that are placed upon non-EU students’ time, such as requirements to take additional licensing exams, secure electives in their home countries, or visa and employment compliance.^26^ These cumulative responsibilities could further restrict the time available for research engagement, making time management more critical for this group. Given the time constraints faced by all medical students in a demanding program, removing barriers to research participation becomes ever more important in supporting their academic and professional goals.

Interestingly, while the majority of students neither agreed nor disagreed, a notable proportion of EU students reported financial constraints as a barrier to research (55.1%). In such cases, the cost of participating in unpaid research, especially if it requires travel or time away from paid employment, can be prohibitive. Although non-EU students face higher tuition fees and reduced access to local grants, some may benefit from international scholarships or strong family support. These findings require further studies to examine in detail and highlight the importance of addressing financial inequities, potentially by providing dedicated stipends or bursaries aimed at supporting student research involvement.

Despite the strengths of this study, several limitations should be considered when interpreting the findings. First, this was a single-centre analysis of one cohort of medical students, which may limit generalisability to the wider Irish and European student populations; further multi-centre studies across diverse cohorts would help address this. Second, the relatively small sample size (n = 114) may be underpowered to fully capture the breadth of student experiences and perceptions regarding research. In addition, the use of a convenience sample introduces the possibility of selection bias. In particular, non-response bias may have arisen if students who did not participate differed systematically from respondents, while self-selection bias is also likely, as students with a pre-existing interest in research may have been more inclined to complete the questionnaire. Furthermore, the reliance on self-reported data introduces the potential for reporting bias, including social desirability and recall bias, which may affect the accuracy of responses. In addition, some survey items contained missing responses. Analyses were performed using available responses for each question, and therefore percentages may not sum to the total study population across all items. The cross-sectional design of the study also limits the ability to infer causality or assess changes in attitudes over time. Additionally, no multivariable adjustment was performed, meaning that potential confounding factors influencing student perceptions were not controlled for. Finally, although the questionnaire was developed to address the study aims, there remains some uncertainty regarding the validity and reliability of the full instrument, which may impact the robustness of the findings.

## Conclusions

This study provides insights into the attitudes, motivations, and perceived barriers to research participation among medical students at an Irish university, with particular attention being paid to differences between students from EU and non-EU countries. Overall, both groups recognise the value of research in medical education and future career development, with minor differences in terms of perceived obligation, motivation, and challenges faced. These findings highlight the importance of tailored, inclusive, and supportive research initiatives that acknowledge students’ diverse needs and backgrounds. By addressing systemic barriers and fostering a culture of peer mentorship and accessibility, medical schools can empower all students to engage meaningfully in research, regardless of their origin or circumstances. Future research with broader sampling and multi-institutional collaboration will be crucial to validating these findings and informing national-level strategies for student research engagement.

## Supplemental Material

Supplemental Material - Perceptions and Attitudes Toward Research Participation Among Medical Students at an Irish Medical School - A Cross-Sectional Study Supplemental Material for Perceptions and Attitudes Toward Research Participation Among Medical Students at an Irish Medical School - A Cross-Sectional Study by Shobha Mehta, Padraig Cronin, Hanru Dong, Maeve Boyle, Mark G. Rae and Mohammed H. Abdulla in Journal of Medical Education and Curricular Development.
