# Supplementary material for: Perceptions and Attitudes Toward Research Participation Among Medical Students at an Irish Medical School - A Cross-Sectional Study
Source: J Med Educ Curric Dev. 2026 Aug 28;13:23821205261466057. doi: 10.1177/23821205261466057 (PMC13527320; doi:10.1177/23821205261466057)
Supplement: Supplementary file 1 [file sj-pdf-1-mde-10.1177_23821205261466057.pdf]

Appendices

Appendix 1. *Study questionnaire*

Q1 Do you consent to participate in this study?

- o Yes (1)
- o No (2)

Q2 Do you consent for your anonymised data to be stored for a minimum of 10 years in UCC?

- o Yes (1)
- o No (2)

Q3 Do you consent for your anonymised data to be posted on an open repository for sharing and collaborative research?

- o Yes (1)
- o No (2)

Q4 Can you please confirm that you are over 18 years of age?

- o Yes (1)
- o No (2)

Q5 What is your sex?

- o Male (1)
- o Female (2)
- o Prefer not to say (3)

Q6 What program are you in?

- o Direct Entry Year 1 (1)
- o Direct Entry Year 2 (2)
- o Direct Entry Year 3 (3)
- o Direct Entry Year 4 (4)
- o Direct Entry Year 5 (5)
- o Graduate Entry Year 1 (6)
- o Graduate Entry Year 2 (7)
- o Graduate Entry Year 3 (8)
- o Graduate Entry Year 4 (9)
- o Other (10)

Q7 What is your highest level of previous education?

- o High School (1)
- o College Degree (2 years) (2)
- o Bachelor's Degree (3)
- o Master's Degree (4)
- o PhD (5)

Q8 Which of the following backgrounds do you come from?

- o Biomedical (1)
- o Non-Biomedical - Sciences (2)
- o Non-Biomedical - Engineering (3)
- o Non-Biomedical - Arts (4)
- o Non-Biomedical - Business (5)
- o Non-Biomedical - Other (6) ____

Q9 Are you an EU or non-EU student?

o EU (1)
o Non-EU (2)

Q10 Do you identify as a racial minority?

o Yes (1)
o No (2)
o I am not sure (3)

Q11 Do you have previous research experience? (including formal academic research, lab volunteering, high school research, etc.)

o Yes (1)
o No (2)

Q12 If yes, please select the areas of research you have experience in from the list below (please select all that apply):

▢ Writing research or grant proposals (1)
▢ Data collection (2)
▢ Data analysis (3)
▢ Manuscript generation or preparation (4)
▢ Wet lab techniques (5)
▢ Research ethics proposals (6)
▢ Animal research (7)
▢ Human trial research (8)
▢ Other (9) ____

Q13 Have you previously published research? (i.e. Journal Article, Oral, Poster, etc.)

o Yes (1)
o No (2)

Q14 What authorship did you hold? (please select all that apply)

▢ First (1)
▢ Second (2)
▢ Last (3)
▢ Other (4)

Q15 How many first author publications?

o 1 (1)
o 2 (2)
o 3 (3)
o 4 (4)
o >4 (5)

Q16 How many second author publications?

o 1 (1)
o 2 (2)
o 3 (3)
o 4 (4)
o >4 (5)

Q17 How many last author publications?

o 1 (1)
o 2 (2)
o 3 (3)
o 4 (4)
o >4 (5)

Q18 How many other author publications?

- o 1 (1)
- o 2 (2)
- o 3 (3)
- o 4 (4)
- o >4 (5)

Q19 Please answer the following questions regarding your perceptions of research:

	Strongly Disagree	Somewhat disagree	Neither agree nor disagree	Somewhat agree	Strongly agree
Research is an important part of medical education ()					
Research is important for my future career ()					
I feel obligated to participate in research activities ()					
Research experience is necessary for my application to internship or residency ()					
I have a genuine interest in research activities ()					
I expect to continue with research activities beyond medical school ()					

Q20 Please answer the following questions regarding any barriers you have perceived to achieving research success:

	Strongly Disagree	Somewhat disagree	Neither agree nor disagree	Somewhat agree	Strongly agree
Limited Finances ()					
Limited Mentorship/Supervision ()					
Limited Research Opportunities ()					
Limited Research Knowledge ()					
Limited Time ()					

Q21 In your opinion, what support would an ideal research mentor provide? (please select all that apply)

- ▢ Networking Opportunities (1)
- ▢ Skills Development (2)
- ▢ Professional Development (CV Building, Interview Advice, etc.) (3)
- ▢ Mentorship/Encouragement (4)
- ▢ Access to Research Opportunities (5)
- ▢ Others: (6) ____

Q22 Feel free to expand on your answers above:

____

Q23 What kind of research activities/workshops would most benefit you? (please select all that apply)

- ▢ Statistical Analysis (1)
- ▢ Academic Writing (2)
- ▢ CV Building (3)
- ▢ xfng Sources (4)
- ▢ Understanding Academic Papers (5)
- ▢ Others: (6) ____

Q24 Feel free to expand on your answers above:

____

Appendix 2. *STROBE Reporting Guideline of observational studies for this paper*

	Item Description	Location (or reason for not reporting)
**Title and abstract**		

1a. Indicate the study's design	Indicate the study's design with a commonly used term in the title or the abstract.	Pg 2
1b. Abstract	Provide in the abstract an informative and balanced summary of what was done and what was found.	Pg 2
**Introduction**		
2. Background / rationale	Explain the scientific background and rationale for the investigation being reported.	Pg 3-5
3. Objectives	State specific objectives, including any prespecified hypotheses.	Pg 5
**Methods**		
4. Study design	Present key elements of study design early in the paper.	Pg 6
5. Setting	Describe the setting, locations, and relevant dates, including periods of recruitment, exposure, follow-up, and data collection.	Pg 6
6a. Eligibility criteria	**Cohort study:** Give the eligibility criteria, and the sources and methods of selection of participants. Describe methods of follow-up. **Case-control study:** Give the eligibility criteria, and the sources and methods of case ascertainment and control selection. Give the rationale for the choice of cases and controls. **Cross-sectional study:** Give the eligibility criteria, and the sources and methods of selection of participants.	Pg 6-7
6b. Matching criteria	**Cohort study:** For matched studies, give matching criteria and number of exposed and unexposed. **Case-control study:** For matched studies, give matching criteria and the number of controls per case.	N/A
7. Variables	Clearly define all outcomes, exposures, predictors, potential confounders, and effect modifiers. Give diagnostic criteria, if applicable.	N/A
8. Data sources / measurement	For each variable of interest give sources of data and details of methods of assessment (measurement). Describe comparability of assessment methods if there is more than one group.	Pg 7
9. Bias	Describe any efforts to address potential sources of bias.	Pg 6 & 7
10. Study size	Explain how the study size was arrived at.	Pg 6 & 7
11. Quantitative	Explain how quantitative variables were handled	Pg 6 & 7

variables	in the analyses. If applicable, describe which groupings were chosen, and why.	
12a. Statistical methods	Describe all statistical methods, including those used to control for confounding.	Pg 6 & 7
12b. Statistical methods – subgroups and interactions	Describe any methods used to examine subgroups and interactions.	Pg 6 & 7
12c. Statistical methods – missing data	Explain how missing data were addressed.	Pg 7
12di. Statistical methods – loss to follow-up	**Cohort study:** If applicable, describe how loss to follow-up was addressed.	N/A
12dii. Statistical methods – matching cases and controls	**Case-control study:** If applicable, explain how matching of cases and controls was addressed.	N/A
12diii. Statistical methods – sampling strategy	**Cross-sectional study:** If applicable, describe analytical methods taking account of sampling strategy.	Pg 7
12e. Statistical methods – sensitivity analyses	Describe any sensitivity analyses.	N/A
**Results**		
13a. Participant numbers	Report the numbers of individuals at each stage of the study—e.g., numbers potentially eligible, examined for eligibility, confirmed eligible, included in the study, completing follow-up, and analysed; Consider use of a flow diagram.	Pg 8
13b. Participants – non-participation	Give reasons for non-participation at each stage.	Pg 8
13c. Participants – flow diagram	Consider use of a flow diagram.	Pg 8
14a. Descriptive data – participant characteristics	Give characteristics of study participants (e.g., demographic, clinical, social) and information on exposures and potential confounders. Present the information in a table.	Pg 9
14b. Descriptive data – missing data	Indicate the number of participants with missing data for each variable of interest.	Pg 11 & 15

14c. Descriptive data – follow-up time	**Cohort study:** Summarise follow-up time—e.g., average and total amount.	N/A
15. Outcome data	**Cohort study:** Report numbers of outcome events or summary measures over time. **Case-control study:** Report numbers in each exposure category, or summary measures of exposure. **Cross-sectional study:** Report numbers of outcome events or summary measures.	N/A
16a. Main results	Give unadjusted estimates and, if applicable, confounder-adjusted estimates and their precision (e.g., 95% confidence intervals). Make clear which confounders were adjusted for and why they were included.	Pg 12 & 16
16b. Main results – category boundaries	Report category boundaries when continuous variables were categorised.	N/A
16c. Main results – risk	If relevant, consider translating estimates of relative risk into absolute risk for a meaningful time period.	N/A
17. Other analyses	Report other analyses done—e.g., analyses of subgroups and interactions, and sensitivity analyses.	N/A
**Discussion**		
18. Key results	Summarise key results with reference to study objectives.	Pg 17
19. Limitations	Discuss limitations of the study, taking into account sources of potential bias or imprecision. Discuss both direction and magnitude of any potential bias.	Pg 20-21
20. Interpretation	Give a cautious overall interpretation considering objectives, limitations, multiplicity of analyses, results from similar studies, and other relevant evidence.	Pg 17-20
21. Generalisability	Discuss the generalisability (external validity) of the study results.	Pg 19-20
**Other information**		
22. Funding	Give the source of funding and the role of the funders for the present study and, if applicable, for the original study on which the present article is based.	N/A
